# Preparation of Fe_3_O_4_/vine shoots derived activated carbon nanocomposite for improved removal of Cr(VI) from aqueous solutions

**DOI:** 10.1038/s41598-023-31015-x

**Published:** 2023-03-09

**Authors:** Maryam Bagherzadeh, Bagher Aslibeiki, Nasser Arsalani

**Affiliations:** 1 Faculty of Physics, University of Tabriz, Tabriz, Iran; 2 Research Laboratory of Polymer, Department of Organic and Biochemistry, Faculty of Chemistry, University of Tabriz, Tabriz, Iran

**Keywords:** Environmental sciences, Natural hazards, Chemistry, Engineering, Materials science, Nanoscience and technology, Physics

## Abstract

In this study, Fe_3_O_4_/activated carbon nanocomposite was successfully synthesized for removal of Chromium from aqueous solutions. The Fe_3_O_4_ nanoparticles were decorated on vine shoots-derived activated carbon using co-precipitation method. The atomic absorption spectrometer was used to evaluate the removal of Chromium ions by the prepared adsorbent. The effect of various parameters such as adsorbent dose, pH, contact time, reusability, electric field, and initial Chromium concentration were examined to find the optimum conditions. According to the results, the synthesized nanocomposite showed a high ability to remove Chromium at an optimized pH of 3. At optimum conditions, a high removal efficiency of 90% and an excellent adsorption capacity of 305.30 mg/g was obtained. In addition, adsorption isotherms and adsorption kinetics were studied in this research. The results showed that the data are well fitted with the Freundlich isotherm and the adsorption process is spontaneous and follows the pseudo-second-order model.

## Introduction

Over the past few years, due to the irreparable damage caused by water pollution, many researchers have begun to remove pollution with various nanocomposites to reduce water pollution^1^. Industrial effluents or sewage, which contain organic or inorganic impurities, such as Chromium, Arsenic, Lead, Nickel, Mercury, Cadmium, etc., are the primary sources of water pollution^2^. Chromium is a primary heavy metal widely used as an occurrence in water bodies from other industrial processes such as leather tanning, plastic, metallurgy industries, wood preservatives, and electroplating which are all examples of industrial pollution^3–6^. As other examples, the release of used Chromium in cooling systems, electroplating industry, tanning industry and paint pigments into the environments increase the toxic effluents, which cause a major issue in food chain^7^. Chromium is a toxic metal and Cr(VI) and Cr(III) are two stable oxidation states of it. Compared with Cr(III), Cr(VI) is much more toxic due to its carcinogenic and mutagenic properties, more soluble, and is more deadly to humans, animals, and plants^8^. To remove Cr(VI), numerous ways, such as ion exchange, chemical reduction, ultrafiltration, adsorption, and biological treatment, have been brought up^9^. According to previous researches, adsorption is cost-effective, efficient, and accessible for removing Chromium (VI) from an aqueous solution^10^. Absorbents can be classified into four groups: biochar-based composite materials, adsorbents, polymers, and activated carbon^11–15^. Due to their high stability and outstanding performances, porous carbon materials have been extensively used in energy storage and water remediation. Among the various types of carbonaceous materials, such as carbon nanofibers, carbon nanotubes, and graphene, activated carbons derived from biomass are incredibly useful due to their environmental and hierarchical structures of the raw material abundance, and low cost^16^. Carbons derived from numerous biomass such as olive pips^17^, palm shells^18^, sugar cane^19^, peanut shells^20^, walnut shells^21^, and persimmon fruit^22^ perform outstandingly in removing pollution from water. The biochar-based composite materials are considered lower cost, better functional, higher efficiency, and more potential adsorption^23^. Presently, biochar-based materials on Cr(VI) removal studies usually focus on magnetic separation and removal efficiency or reusability. In synthesizing magnetic adsorbents, magnetite nanoparticles are typically used as a magnetic material to separate metal ions and organic pollutants. In this study, Fe_3_O_4_/C was prepared in two stages, including synthesizing magnetic nanoparticles and their modification using activated carbon. The present work investigates the ability of the activated carbon derived from the vine shoots to remove Cr(IV) ions, considered one of the most toxic heavy metals produced in industrial technologies, causing significant environmental and economic problems from the water. We focused on enhancing the adsorption capacity and efficiency using Fe_3_O_4_ magnetic nanoparticles grafted with activated carbon (Fe_3_O_4_/C) nanocomposite as an adsorbent to remove Chromium from the aqueous solution. Moreover, we discussed the adsorption behaviors using batch experiments and related physical and chemical mechanisms. In addition to investigating the effects of various parameters affecting adsorption, the effect of electric field on adsorption efficiency and recyclability of the Fe_3_O_4_/C composite was also assessed. The preparing method and synergistic interactions could be a generalized strategy for the nanoparticle/ porous materials system. The improved adsorption performance may apply to environmental protection and sustainable resources.

## Results and discussion

### Morphology and structure of the composite

**Figure 1:**
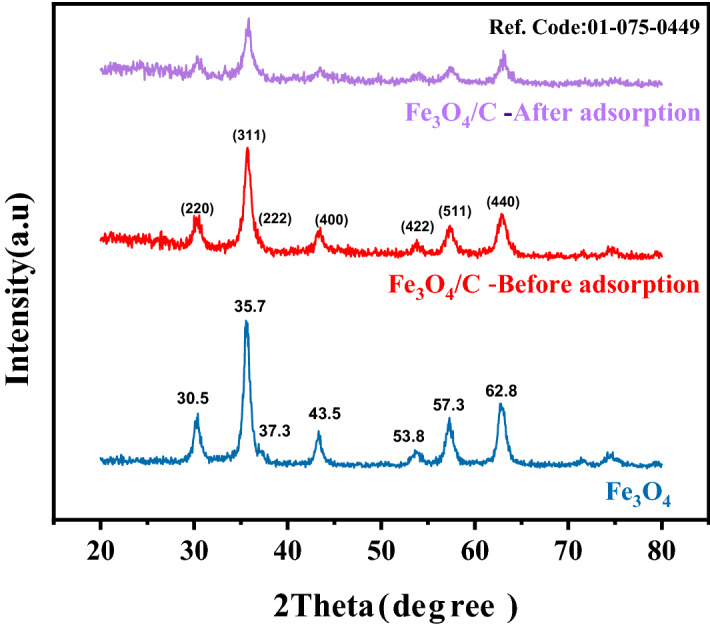
XRD patterns of Fe_3_O_4_ and Fe_3_O_4_/C before adsorption, Fe_3_O_4_/C after adsorption samples.

The results of the XRD for Fe_3_O_4_ nanoparticles and the Fe_3_O_4_/C magnetic composite in the range of 2θ = 20–80° are shown in Fig. 1. The diffraction patterns of the samples have peaks at 30.05, 35.07, 37.30, 43.05, 53.80, 57.03, and 62.80°, corresponding to the (220), (311), (222), (400), (422), (511) and (440), lattice planes of the FCC structure of Fe_3_O_4_ phase (JCPDS card No.96-900-5843), respectively. There are no significant structural change in XRD pattern of nanocomposite, before and after adsorption process, which reveals structural stability of the prepared sample.

**Figure 2:**
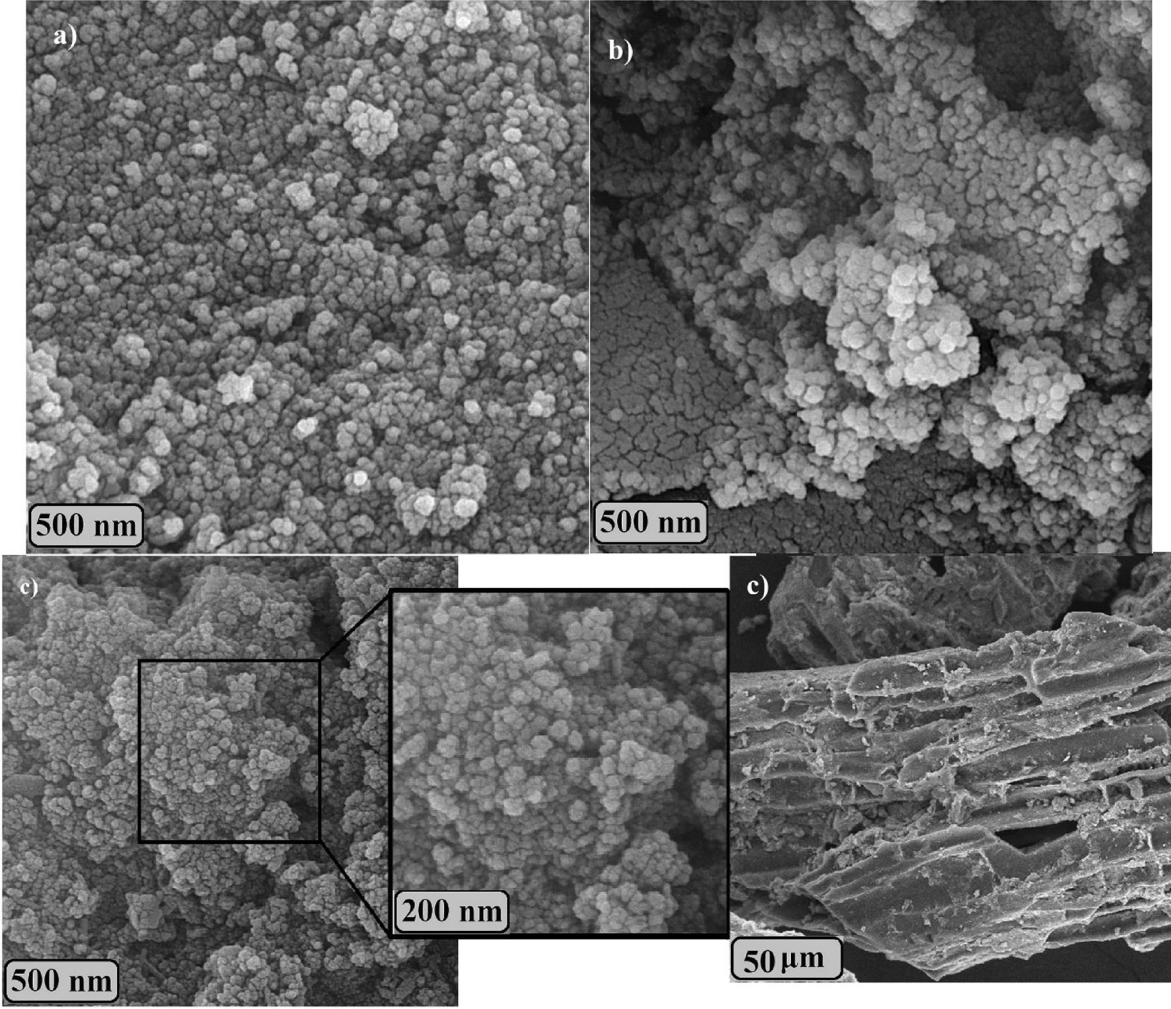
SEM images of (**a**) Fe_3_O_4_, and Fe_3_O_4_/C, (**b**) before and (**c**) after Cr(VI) adsorption.

The FE-SEM was used to evaluate and compare the morphology of the synthesized samples. Figure 2 shows the SEM images of the samples. As can be seen in Fig. 2a, the Fe_3_O_4_ sample consists of magnetic nanoparticles. After the preparation of nanocomposite (Fe_3_O_4_/C) the morphology (Fig. 2b) has become more uniform in comparison to Fe_3_O_4_ sample. In the case of activated carbon nanocomposites, carbon penetrates the cavities between the catalysts and creates a uniform surface. In addition, one of the criteria for the proper performance of nanoparticles is the lack of particle aggregation, which was not observed in these nanostructures, indicating their successful synthesis. As exhibited in the image, the adsorbent has a spherical structure, and after modifying it with activated carbon, the adsorbent has maintained its spherical structure. The SEM image for adsorbent after adsorption process (Fig. 2c) confirms morphological stability of the prepared sample.

**Figure 3:**
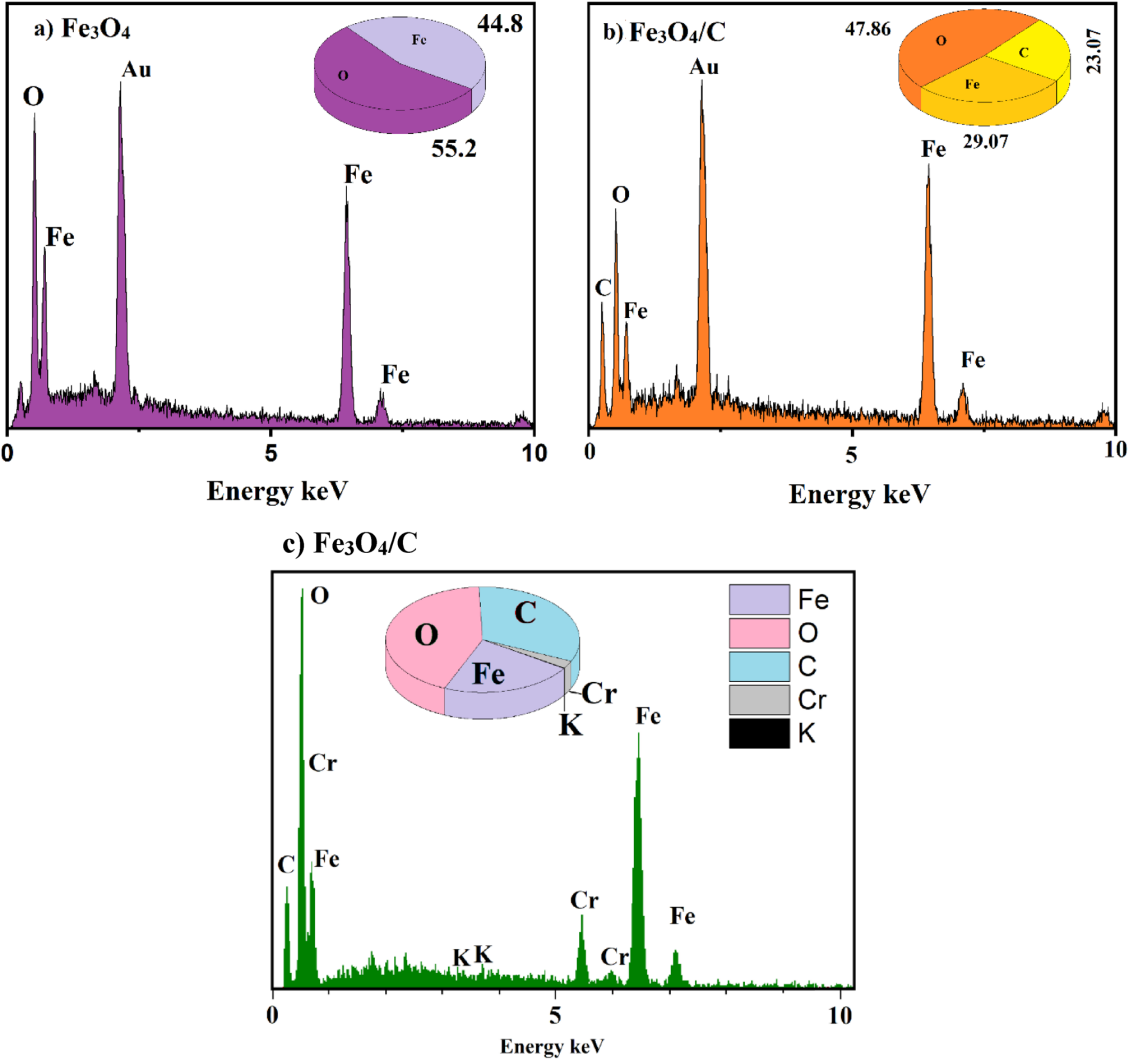
EDX spectrum (**a**) Fe_3_O_4_, (**b**) Fe_3_O_4_/C before adsorption, (**c**) Fe_3_O_4_/C after adsorption Cr(VI) adsorption.

**Figure 4:**
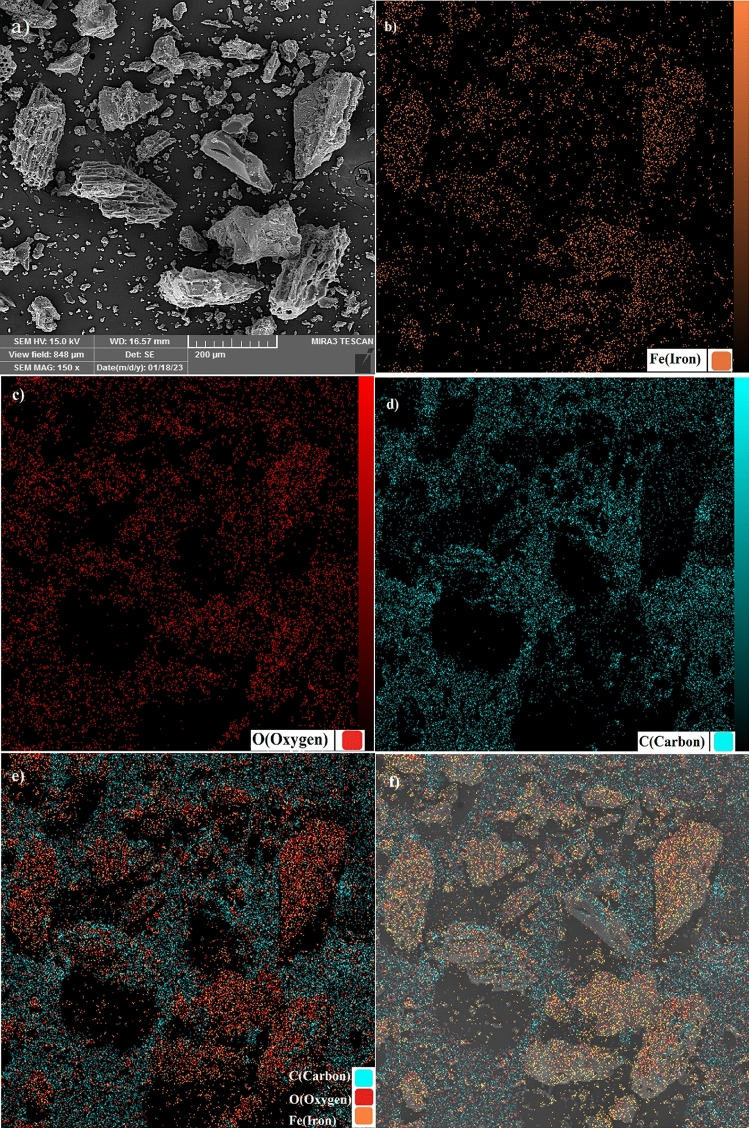
Elemental mapping analysis of Fe_3_O_4_/C nanocomposite.

EDX analysis was used to investigate the chemical elements of the samples. The results of this analysis are shown in Fig. 3. The figure shows that the iron, oxygen, and carbon elements are present in the powders. By calculating stoichiometry from the chemical formula, the experimental and stoichiometric values are almost in good agreement with each other. Elemental mapping analysis shows the presence and dispersion of the iron in the Fe_3_O_4_/C nanocomposite (Fig. 4). This analysis confirms that the adsorbent was fully coated with magnetite and had no free activated carbon.

**Figure 5:**
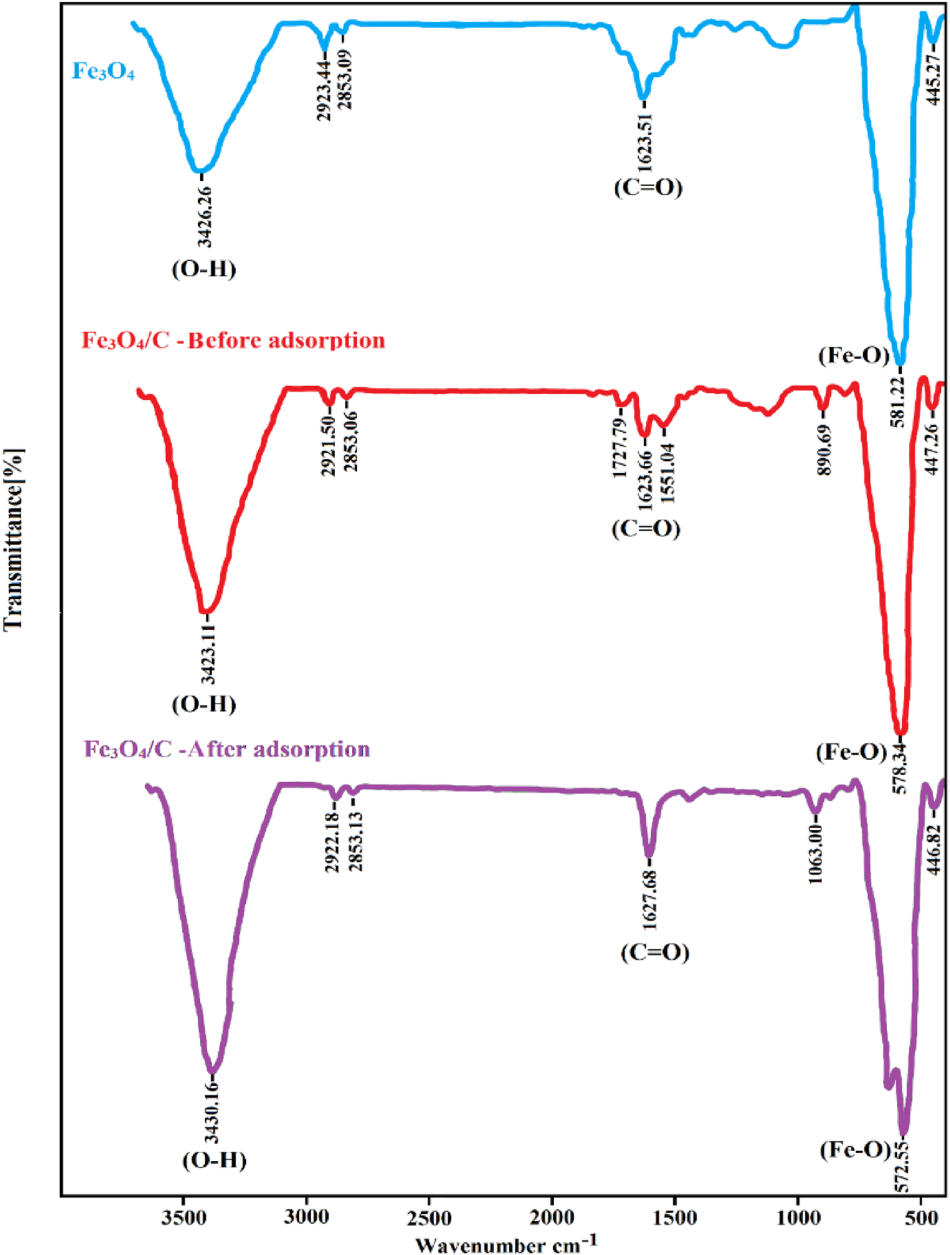
FT-IR spectra of Fe_3_O_4_ and Fe_3_O_4_/C before adsorption, Fe_3_O_4_/C after adsorption samples.

Figure 5 shows the Fourier transform infrared (FT-IR) spectra of the samples. In the FT-IR spectra of Fe_3_O_4_, the characteristic peaks at 581 cm^−1^ were assigned to Fe–O in the spinel phase. The peaks observed in 3426 and 1623 cm^−1^, corresponding to vibrations of (OH) and (C=O) stretching bonds, respectively. In addition, the peaks in 2853 and 2923 cm^−1^ are related to aldehydes’ stretching vibrations (–C–H). In the Fe_3_O_4_/C nanocomposite spectra, there are two peaks at 1551 and 1623 cm^−1^, indicating the carbonyl group for the amides. Also, the sharpening of the 3423 cm^-−1^ peak is due to the overlap related to vibrations of OH and (N–H) stretching. The peak of flexural vibrations -NH was observed in 2921 and 2853 cm^−1^, which belongs to the amide functional group and overlaps with the stretching vibrations –C–H of the aldehyde group. Therefore, there is a slight difference compared to the peaks of the Fe_3_O_4_ spectra.

**Figure 6:**
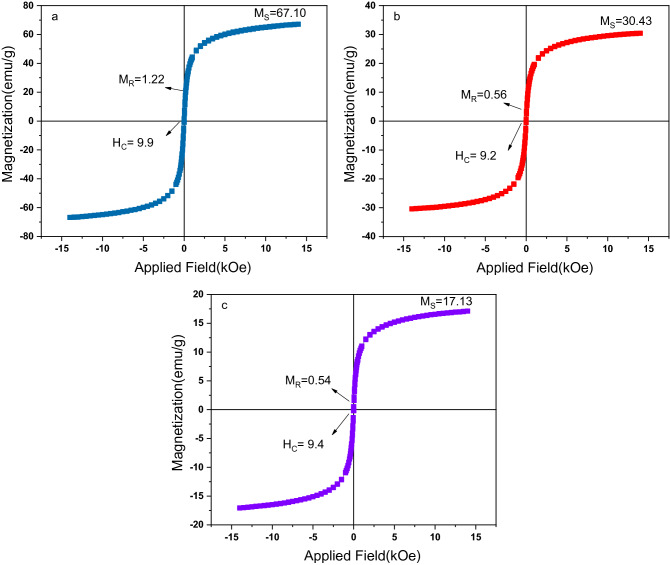
Magnetic hysteresis loops of (**a**) Fe_3_O_4_, (**b**) Fe_3_O_4_/C, before adsorption, and (**c**) Fe_3_O_4_/C after adsorption.

According to the VSM results in Table 1 and Fig. 6, it can be seen that the maximum magnetization of Fe_3_O_4_ is higher than Fe_3_O_4_/C nanocomposites, which is due to non-magnetic nature of the activated carbon. The coercive field of iron ferrite is almost equal to that of the composite sample. The M_R_/M_Max_ ratio is also constant as the residual magnetization decreases. However, the magnetic property of Fe_3_O_4_/C is strong enough that it can to be quickly separated from the heavy metal ion solution using an external magnetic field. Also the result shows that both samples are superparamagnetic; also composite saturation magnetization of 30.34 emu g^−1^, gives the adsorbent the privilege of magnetic separation and good retrievability. The observed reduction in magnetization of composite sample after adsorption could be due to adsorbed moisture and presence of nonmagnetic Cr(VI) ions.

**Table 1:** Magnetic parameters measured by VSM.

Samples	M_Max_ (emu/g)	M_R_ (emu/g)	H_C_(O_e_)	M_R_/M_Max_
Fe_3_O_4_	67.10	1.22	9.9	0.018
Fe_3_O_4_/C before adsorption	30.34	0.56	9.2	0.018
Fe_3_O_4_/C after adsorption	17.13	0.54	9.4	0.031

**Figure 7:**
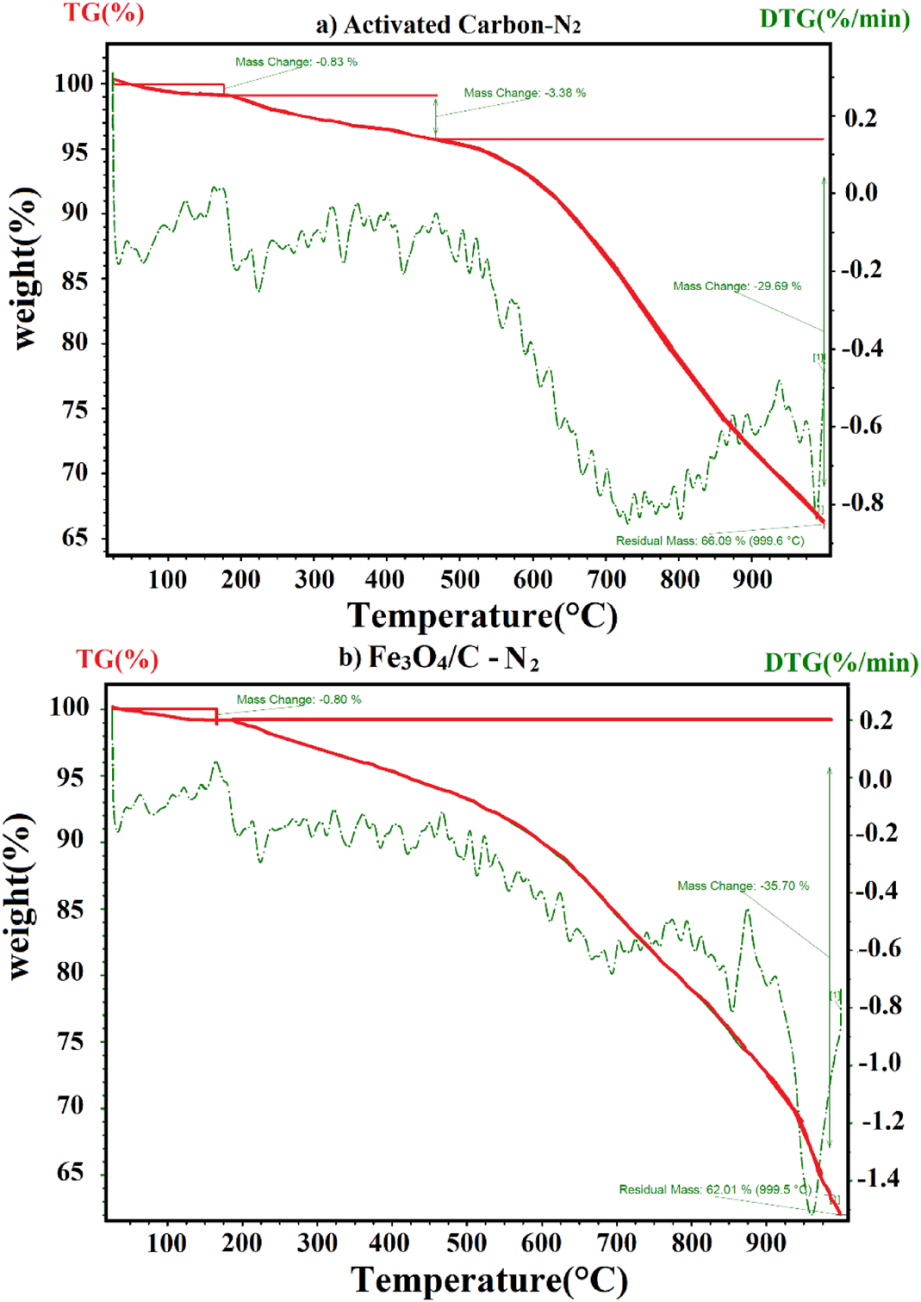
Thermogravimetric analysis (TGA) of (**a**) activated carbon, (**b**) Fe_3_O_4_/C.

Figure 7 shows the TGA and corresponding DTG curves of the activated carbon and Fe_3_O_4_/C. For the activated carbon sample, a three-step degradation pattern was observed with the first step upon increasing the temperature to 175 °C, the weight of the sample was reduced by 0.83%, apparently caused by the loss in moisture content followed by a 3.38% reduction in weight between 175 and 475 °C in the second-step which may be attributed to loss of hemicellulose and cellulose residues as well as volatile fractions. In the third step, at 475–999 °C a much higher loss in weight by 29.69% was observed, accounting for the devolatilization of thermally stable volatile compounds, degradation of lignin and oxidation of carbon.

As an activation temperature of 700 °C for 1.5 h was used for preparation of AC, most of the hemicellulose and cellulose as well as volatile fractions should undergo decomposition resulting in only a minor loss 3.38% in the second step. Also observed weight-loss pattern for Fe_3_O_4_/C nanocomposite, shows weight reduction of 0.80%, and 35.70% for the temperature ranging from 25 to 175 °C and 175 to 999 °C, respectively.

### Adsorption of Cr(VI) by Fe_3_O_4_/C nanocomposite

The stock solution (500 mg/L) was prepared by dissolving 1.414 g potassium dichromate in 1 L deionized water, and 50 mg/L concentration was prepared by diluting the stock solution. To find the optimal pH in the absorption of hexavalent chromium by Fe_3_O_4_/C nanocomposite, 15 mL chromium solutions with a concentration of 50 mg/L were used at different pHs (2, 3, 4, 6, 8, 10, and 12). 0.1 M sodium hydroxide solution and 0.1 M hydrochloric acid were used to adjust the initial pH of the solutions in this range, and finally, pH = 3 was chosen as the optimal pH.

The effect of contact time between chromium solution and adsorbent was investigated at different contact times of 1, 5, 15, 30, 60, 90, and 120 min in optimal pH with a concentration of 50 mg/L. Therefore, to check other absorption parameters, 30 min time was considered to reach equilibrium. Because of increasing the contact time from the beginning of the absorption process until 30 min, the amount of absorption increased from 42 to 62%, while from 30 to 120 min.

To determine the optimal amount of adsorbent dose for absorbing Cr(VI) from a solution (15 mL) with a concentration of 50 mg/L, different amounts (2–10 mg) of synthesized Fe_3_O_4_/C adsorbent were added to potassium dichromate solutions. Then the chromium solutions containing the corresponding adsorbent were stirred by a mechanical shaker. After the optimal period of 30 min, the samples were absorbed by an external magnet. The optimal value of adsorbent dose for Cr(VI) removal by Fe_3_O_4_/C nanocomposite was selected as 6 mg.

In order to study the effect of concentration change on chromium removal, solutions with different initial concentrations (5, 25, 75, 100, 150, 200 mg/L) of chromium were prepared. The removal of hexavalent chromium in this range of different concentrations was investigated by 6 mg Fe_3_O_4_/C adsorbent at pH = 3 and at room temperature. These solutions were stirred by a mechanical shaker for an optimal period of 30 min.

### Effect of pH on Cr(VI) removal

**Figure 8:**
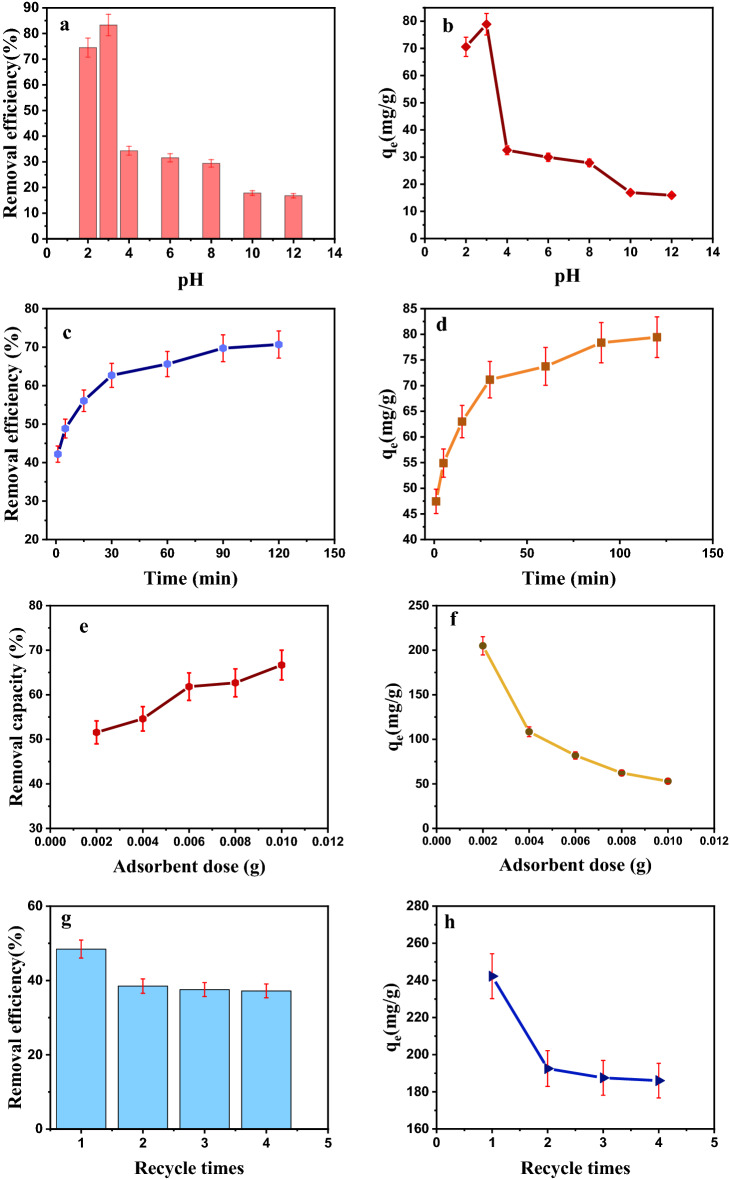
The effect of different parameters on Cr(VI) removal.

pH is one of the critical parameters that affects the adsorption surface load and contaminant structure in the adsorption process. In this study, the removal of Cr(VI) was studied in batch experiments at the pH = 2, 3, 4, 6, 8, 10, and 12. The experiments were performed using adsorbent dose of 0.007 g, the initial concentration of 50 mg /L, the contact time of 30 min and ambient temperature of 25 ± 2 °C (Fig. 8a,b). Due to the additional OH^−^ ions and a negative charge at alkaline pHs, the adsorbent surface had a negative charge; thus, a repulsive force was created between the adsorbent and negatively charged Chromium molecules. Subsequently, the adsorption efficiency will be reduced. But at acidic pHs, the Fe_3_O_4_/C adsorbent surface will have a positive charge because increasing the production of available protons can add to the adsorbent surface, creating an electrostatic attraction between the adsorbent and the adsorbate and ultimately increasing the adsorption efficiency. The efficiency of Cr(VI) adsorption decreases by increasing pH. The decreasing trend might be attributable to the changes in Chromium speciation and surface charges of the adsorbent as a function of pH. Cr(VI) shows different oxidation states In aqueous solutions, and it commonly occurs in one of three ionic forms, i.e., dichromate $\left({\mathrm{Cr}}_{2}{\mathrm{O}}_{7}^{-}\right)$, bichromate $\left({\mathrm{HcrO}}_{4}^{-}\right)$, and chromate $\left({\mathrm{CrO}}_{4}^{2-}\right.$). In addition, the concentration of OH^−^ increases, which compete with $\left({\mathrm{Cr}}_{2}{\mathrm{O}}_{7}^{-}\right)$, and $\left({\mathrm{CrO}}_{4}^{2-}\right.$) ions to be adsorbed on the surface of Fe_3_O_4_/C NPs. Thus, the removal efficiency decreases^24^.

### The effect of contact time on Cr(VI) removal

Contact time is one of the most critical factors affecting the absorption process. Because the adsorption process is equilibrium, the contact time factor represents the equilibrium time between the adsorbent and the adsorbate and plays an essential role in the reaction. Therefore, to investigate the effect of contact time on the adsorption process, the relevant experiments were performed at pH = 3, and different contact times of 1, 5, 15, 30, 60, 90, and 120 min. The adsorbent dose was 0.007 g and a concentration of was 50 mg/L. The Chromium removal rate in the first 60 min of absorption was much faster than in the next 60 min and gradually slowed down. One of the reasons that can increase the adsorption rate at the beginning of the process is the presence of active sites on the adsorbent surface. The number of these active sites is higher at first also the solute concentration gradient is high. Still, over time and by occupying them, a repulsive force is created between the molecules dissolved in the bulk and solid phase, which leads to a decrease in adsorption efficiency. This shows the possible monolayer formation of Cr(VI) ions on the outer surface^25^. It is evident from the Fig. 8c,d that more than 90% of removal of Cr takes place within 30 min.

### The effect of adsorbent dose on Cr(VI) removal

Increasing the adsorbent dose provides more exchange surface to the adsorbent, increasing the equilibrium absorption. Thus, solutions with a concentration of 50 mg/L and pH = 3 (optimal) and a contact time of 30 min (optimal) with different amounts of absorbent dose (0.002, 0.004, 0.006, 0.008, 0.01 g) were used. As shown in Fig. 8e,f, the amount of Chromium adsorption increases with increasing adsorbent dose because the number of active adsorption sites on the adsorbent surface and empty sites increases with an increase the amount of adsorbent. The increased amount of adsorbent positively and negatively affected adsorption efficiency and capacity, respectively. The saturation of active sites on the adsorbent surface during the adsorption process was probably due to the decreased Chromium adsorption rate with increasing Fe_3_O_4_/C adsorbent concentration. Increasing the free sites on the adsorbent surface is the main reason for increasing the adsorption efficiency by increasing the adsorbent dosage. Thus, with increasing concentration, the ratio of Chromium concentration to the available surface of the adsorbent decreases. As a result, the adsorption efficiency of Chromium decreases. Due to the reduction of the surface available for absorption and the agglomeration of the absorption area at higher doses, the rate of adsorption reaches saturation at higher doses. Therefore, a large amount of nanocomposite Fe_3_O_4_/C can purify a larger amount of water solution with a certain amount of adsorbent^26^.

### The effect of reusability on Cr(VI) removal

To stabilize the adsorption process, the adsorbents must be reusable. Studies have been performed to evaluate the reuse potential of nanocomposites. In this research, the adsorbent dose was 0.006 g, pH was 3, initial concentration was 200 mg/L, and contact time was 30 min at room temperature. According to the results obtained and shown in Fig. 8g,h, the adsorption efficiency of the first load compared to the second load has decreased by only 10%. Next, only a 1% decrease in adsorption efficiency is observed, indicating the adsorbent’s stability.

### The effect of electric field on Cr(VI) removal

**Figure 9:**
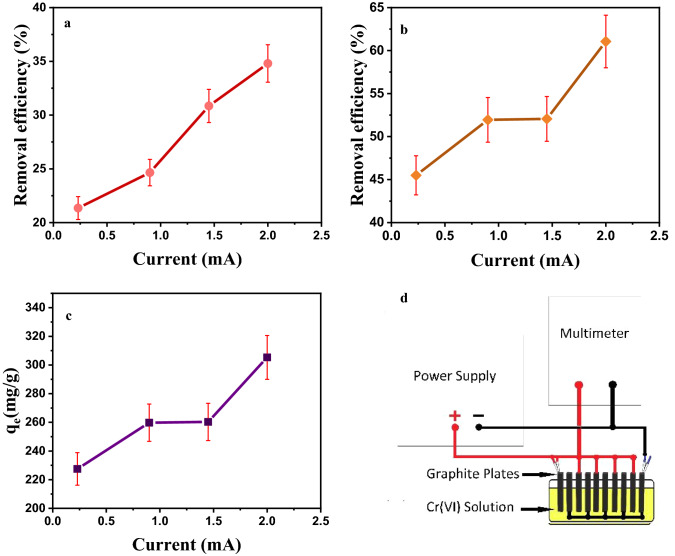
Effect of electric field on Chromium adsorption efficiency. (**a**) Without adsorbent, (**b**) after adding the adsorbent, (**c**) adsorption capacity, (**d**) schematic illustration of the set-up.

The effect of electric field is one of the parameters that was investigated in this research. We used the idea of electrokinetic soil remediation using electric field^27^. Applying an electric field can increse the ions movements inside the solution. The applied electric force causes accumulation, precipitation and chemisorption of ions on graphitic cathodes, hence increse the removal efficiency^28^. In this section, 10 rectangular graphite electrodes were used, located at the same distance, and connected to the voltage supply as cathode and anode (Fig. 9d). The initial concentration of Chromium was 200 mg/L, and pH = 3. Voltages equal to (5, 10, 15, 20 V) were applied with currents (0.23, 0.90, 1.45, 2 mA) for 10 min. After applying the voltage, 0.006 g of adsorbent was added to the solution, and a shaker stirred it for 30 min. Figure 9a,b show the effect of electric field on Chromium adsorption efficiency without using adsorbent and after adding the adsorbent, respectively. In addition, the Fig. 9c shows adsorption capacity in the presence of electric field. It is clear from the figure that the q can reach to a high value above 300 mg/g by applying a small electric field.

### The effect of initial Cr(VI) concentration

The effect of initial Cr(VI) concentration on adsorption, adsorbent dose 0.006 g, and initial Chromium concentrations (5, 25, 75, 100, 150, 200 mg /L) and other parameters were constant. The amount of residual Chromium was determined in solution after 30 min. It can be inferred that with increasing the initial concentration of Chromium, the number of active adsorption sites at the adsorbent surface fills faster; as a result, the adsorption efficiency decreases.

### The effect of initial Cr(VI) concentration on adsorption isotherms

**Figure 10:**
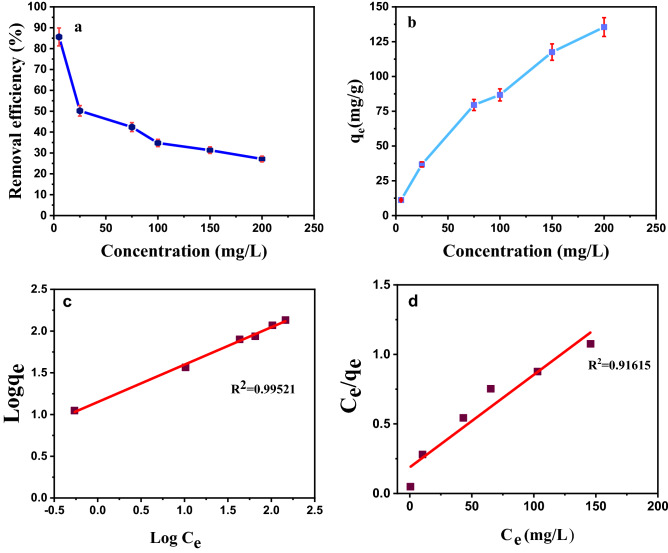
Effect of initial Cr(VI) concentration. (**a**) removal efficiency, (**b**) adsorption capacity, (**c**) Freundlich isotherm plot, (**d**) Langmuir isotherm plot for adsorption of Cr(VI) using Fe_3_O_4_/C.

Figure 10 shows the effect of initial Cr(VI) concentration on removal efficiency and adsorption capacity. Different models and equations of adsorption equilibrium isotherms are used to describe the adsorbent surface properties, the maximum adsorption amount, and conditions for optimal adsorption^29^. For this purpose, the Langmuir and Freundlich isotherms have been used in this study. Figures 10c,d show Langmuir and Freundlich isotherms plot for adsorption of Cr(VI) using Fe_3_O_4_/C. The Langmuir isotherm model is based on the uniform adsorption of adsorbent material with the same energy on all adsorbent surfaces. The Freundlich isotherm is based on the multilayer and heterogeneous adsorption of the adsorbent on the adsorbent. The effect of the initial concentration of the Cr(VI) was investigated in the range of 5–200 mg/L with the pH of 3. The fit results by two models reveals that the experimental data were in accordance with Freundlich isotherm, Table 2. Freundlich model shows that multilayer adsorption was carried on the adsorbent surface. be used the value of the n parameter in the Freundlich isotherm (which is greater than one) indicates the suitability of Cr(VI) adsorption to the Fe_3_O_4_/AC^30^.

**Table 2:** Langmuir isotherm and Freundlich isotherm constants for Cr(VI) adsorption by Fe_3_O_4_/C.

Langmuir				Freundlich		
K_L_ (L/mg)	q_m_ (mg/g)	R_L_	R^2^	n	K_F_ (L/g)	R^2^
28.94	5.31	0.00046	0.91615	2.23	14.11	0.99521

### The N_2_ adsorption and desorption isotherms

**Figure 11:**
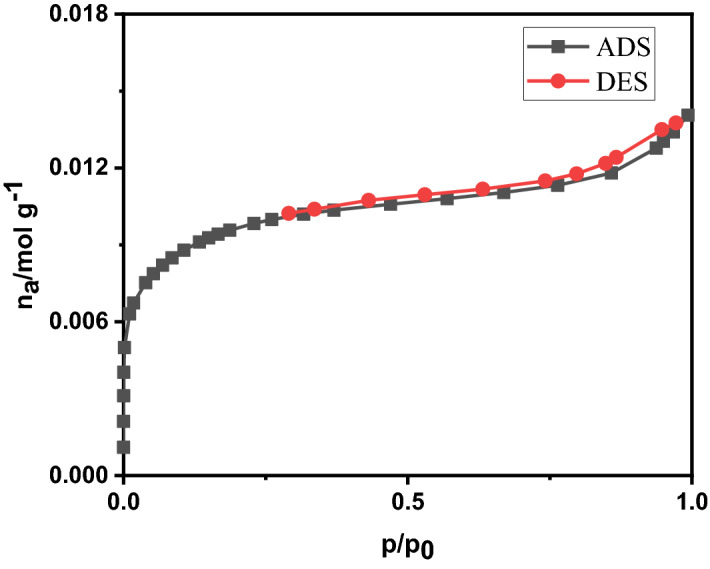
The N_2_ adsorption–desorption isotherm.

The N_2_ adsorption–desorption isotherm of Fe_3_O_4_/C is given in Fig. 11, related to the adsorption isotherm of type IV. This type of isotherm is for mesoporous materials^31^, and is often observed for catalysts, and the corresponding curve is used to determine the pore size distribution. Hysteresis indicates the presence of mesoporous pores in the material. The smaller pores are related to the carbon deposition on the surface of nanoparticles; larger mesopores may form between secondary aggregated particles. The mesopores and their high surface area endow the as-prepared Fe_3_O_4_/C with novel adsorption application potentials.

### The effect of temperature on Cr(VI) removal

**Figure 12:**
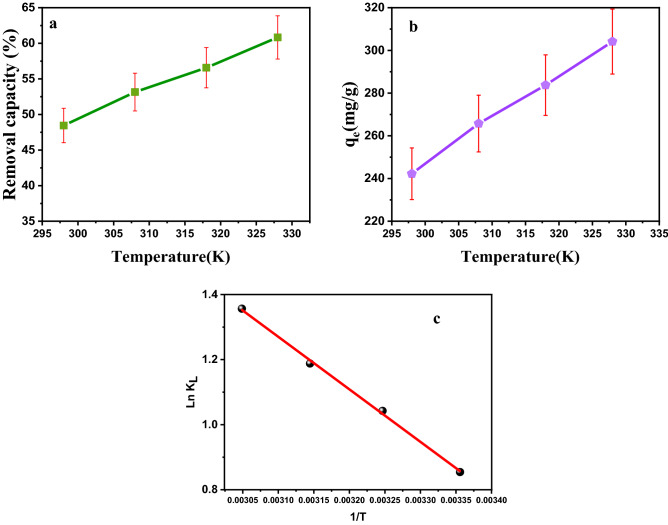
Effects of temperature on Cr(VI) removal by the Fe_3_O_4_/C (**a**) removal efficiency, (**b**) adsorption capacity, (**c**) Ln K_e_ plot 1/T for Chromium adsorption.

In this part, the adsorbent dose was 0.006 g, the initial concentration was 200 mg/L, pH = 3, contact time of 30 min, and temperatures of bath were 298, 308, 318 and 328 K. According to the obtained results in Fig. 12, it is observed that with increasing temperature, the percentage of adsorption efficiency and adsorption capacity increase. In the study of adsorption processes, another important factor is the use of thermodynamic parameters of adsorption. It is necessary to determine the changes of three standard enthalpy factors ($\Delta {\mathrm{H}}^{\circ }$), free energy ($\Delta {\mathrm{G}}^{\circ }$), and standard entropy ($\Delta {\mathrm{S}}^{\circ }$). Regardless of the adsorption process being endothermic or exothermic, as well as to determine the spontaneity of the reaction. Using the following equations, these parameters are calculated. Figure 12, and Table 3 show the relevant parameters^32^:

**Table 3:** Thermodynamic parameters for Cr(VI) adsorption.

Temperature (K)	K_e_	$\Delta {\mathrm{G}}^{\circ }$(kJ mol^−1^)		$\Delta {\mathrm{H}}^{\circ }$ (KJ/mol)	$\Delta {\mathrm{S}}^{\circ }$ (J/K mol)
		$\Delta {\mathrm{G}}^{\circ }=-\mathrm{RT Ln}{\mathrm{K}}_{\mathrm{e}}$	$\Delta {\mathrm{G}}^{\circ }=\Delta {\mathrm{H}}^{\circ }-\mathrm{T\Delta }{\mathrm{S}}^{\circ }$		
298	2.34	− 2.11	− 2.12	13.42	52.18
308	2.38	− 2.66	− 2.64		
318	3.28	− 3.14	− 3.16		
328	3.88	− 3.69	− 3.68		

1 $${\text{K}}_{{\text{e}}} = \frac{{{\text{C}}_{{{\text{Ae}}}} }}{{{\text{C}}_{{\text{e}}} }}$$

2 $${\mathrm{LnK}}_{\mathrm{e}}=\frac{\Delta {\mathrm{S}}^{\circ }}{\mathrm{R}}-\frac{\Delta {\mathrm{H}}^{\circ }}{\mathrm{RT}}.$$

3 $$\Delta {\mathrm{G}}^{\circ }=\Delta {\mathrm{H}}^{\circ }-\mathrm{T\Delta }{\mathrm{S}}^{\circ }$$

According to the results of Table 3, it can be seen that the values ($\Delta {\mathrm{G}}^{\circ }$) in the two equations of $\Delta {\mathrm{G}}^{\circ }=-\mathrm{RT Ln}{\mathrm{K}}_{\mathrm{e}}$ and $\Delta {\mathrm{G}}^{\circ }=\Delta {\mathrm{H}}^{\circ }-\mathrm{T\Delta }{\mathrm{S}}^{\circ }$ are slightly different.

### Adsorption kinetics

Kinetic equations are used to investigate the factors affecting the reaction rate. We used pseudo-first-order and pseudo-second-order kinetic models that are more common for the Chromium adsorption process^33,34^. The kinetics of sorption can be expressed by a first-order model, which leads to the following equation:

4 $$\mathrm{ln}\left({q}_{\mathrm{e}}-{q}_{\mathrm{t}}\right)=\mathrm{ln}{q}_{\mathrm{e}}-{k}_{1}t$$

where q_e_ and q_t_ (both in mg/g) are the removal amounts of Cr(VI) at equilibrium time and at time t (min), respectively. The k_1_ (min^−1^) is the corresponding adsorption rate constant. The values of q_e_ and k_1_ are determined from the intercept and the slope of the plots of ln (q_e_ − q_t_) versus t, respectively.

**Figure 13:**
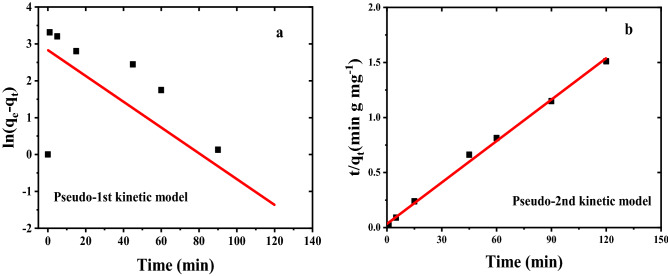
(**a**) First-order, (**b**) second-order plot for adsorption of Cr(VI) by Fe_3_O_4_/C nanocomposite.

The pseudo-second-order can be represented as follows:

5 $$\frac{\mathrm{t}}{{\mathrm{q}}_{\mathrm{t}}}=\frac{1}{{\mathrm{K}}_{2}{\mathrm{q}}_{\mathrm{e}}^{2}}+\frac{t}{{\mathrm{q}}_{\mathrm{e}}}$$

where K_2_ is the pseudo-second-order rate constant. The kinetics of the Cr(VI)/Fe_3_O_4_/C system were analyzed at initial pH values of 3. Figure 13 shows the non-linear experimental data to the kinetic models. The magnitude of R^2^ in Table 4 indicated that the adsorption of Cr(VI) onto the sorbent was best fitted with a pseudo-second-order kinetic model.

**Table 4:** Kinetic parameters for Cr(VI) adsorption.

Parameter	Value
V_m_ (cm^3^(STP)/g)	174
a_s_, _BET_ (m^2^/g)	759
*C*	1088.8
Total pore volume (p/p_0_ = 0.990) (cm^3^/g)	0.4848
Mean pore diameter (nm)	2.5536

According to the values obtained from the kinetic parameters of Chromium adsorption by Fe_3_O_4_/C nanocomposite, the calculated adsorption capacity values (q_e_.Cal) in the pseudo-second-order kinetic model are 79.74 mg/g, which is closer to the adsorption capacity of the experiments (q_e_.exp = 79.43 mg/g). The adsorption capacity in the pseudo-first-order model is 16.99 mg/g. By comparing the correlation coefficient (R^2^) of the two kinetic models, it is concluded that the adsorbent follows the pseudo-second-order kinetic. The difference in the reports can be caused by the difference in the conditions of the adsorption tests and the type of adsorbents^35^.

### BET analysis

**Figure 14:**
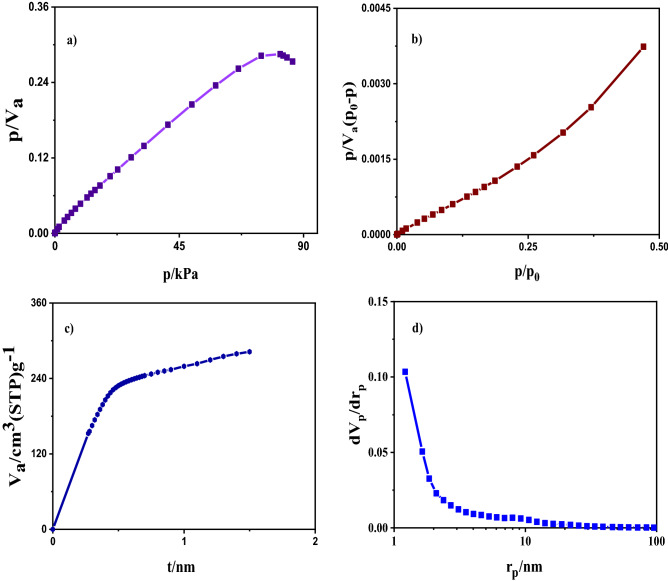
(**a**) Langmuir plot, (**b**) BET plot, (**c**) t plot, (**d**) BJH plot.

Knowing the specific surface of the absorbent is considered one of the essential factors for the adsorbent. The higher the specific surface area, the greater the porosity of the material and the greater the contact surface with the adsorbing material. The results are presented in Table 5 and Fig. 14. The specific surface obtained from this analysis is 759 (m^2^/g). And the total volume of porosity V_m_ is 174 (cm^3^(STP)/g). The mean pore diameter is 2.5 nm. To measure micropores in the presence of mesoporous, t plot analysis is used, which is usually used to measure the volume and surface area of micropores. The specific surface area of all mesoporous and micropores is a_1_ = 871.3 (m^2^/g), the volume of absorbed gas in standard conditions is V_1_ = 0 (cm^3^/g), and its linear slope is 565/41. Pore size distribution is calculated through the BJH diagram, which has a ascending trend from 1.2 to 8 nm. The diameter of the pore, the regional distribution of the pores, and the volume of the pores are obtained from this method. This method is effective for the scale area of macropore and small mesoporous. The distribution of the size of the pores is based on their radius, the highest frequency of which is 1.21 nm. The total pore volume is V_p_ = 0.218 (cm^3^/g) and the obtained specific surface is a_p_ = 166.56 (m^2^/g). Comparable BET analysis some related materials reported by others – for instance, the magnetic carbon materials synthesized by (i) Fe_3_O_4_/C from termite feces as a precursor with S_BET_ values of 699(m^2^/g). The pore size distribution radius is about 1.8–2.5 nm. The shape of the holes is cylindrical they can be classified as type IV, characteristic of mesoporous material^30^. (ii) Surface area, pore volume, particle size, and average pore diameter of Fe_3_O_4_/C (Sunflower Head Waste) are 51.1 (m^2^/g), 0.0386 (cm^3^ g^−1^), 9 to 18 nm and 3.293 Å, respectively. The nanoparticles shape and structure are tubular and porous^36^. (iii) (Fe-MCNs) magnetic mesoporous carbon nanospheres with pore size, S_BET_ and pore volume obtained are 2.7 nm, 619 (m^2^/g) and 0.52 cm^3^ g^−1^, respectively and the shape of Fe-MCNs are spherical with diameters of ~ 110 nm^37^.

**Table 5:** Textural characteristics of the prepared Fe_3_O_4_/C.

Pseudo-first-order			Pseudo-second-order			
q_e.cal_ (mg/g)	K_1_ (1/min)	R^2^	q_e.exp_ (mg/g)	K_2_ (g/mg.min)	q_e.cal_ (mg/g)	R^2^
16.99	− 0.00029	0.49432	79.43	0.005271	79.74	0.99592

### Comparison of Fe_3_O_4_/C with other adsorbents

The maximum adsorption capacity of Fe_3_O_4_/C sorbent for Cr(VI) removal was compared with that of various other adsorbents. The results are presented in Tables 6 and 7. As shown in the Table 6, the adsorption capacity of Fe_3_O_4_/C sorbent for Chromium removal is much higher (305.30 mg/g) than previously reported using other adsorbents. These results confirm the high potential of the prepared nanocomposite for wastewater treatment applications and heavy metals removal.

**Table 6:** Comparison of Cr(VI) adsorption performance of various activated carbons.

Adsorbent (activated carbon)	pH	Maximum adsorption capacity (mg/g)	Ref
Pinecone	3.0	7.48	^38^
*Posidonia oceanica* seagrass	3.0	118.00	^32^
*Ziziphus jujuba* cores	2.0	124.25	^39^
Eucalyptus sawdust	2.0	12.00	^40^
*Aegle marmelos* fruit shell	2.0	43.54	^41^
Luffa sponge	1.0	149.06	^42^
Chestnut oak shells	3.0	85.47	^43^
Date seeds	2.0	42.57	^44^
Nutshell	2.0	43.45	^3^
Sunflower head waste	2.0	4.40	^45^
Longan seed	3.0	35.02	^36^
Fertilizer industry waste	2.0	15.24	^46^
Vine shoots	3.0	305.30	This study

**Table 7:** Analogy of various Fe_3_O_4_/C adsorbent characterization for the Cr(VI) and heavy metalsremoval.

Adsorbent (Fe_3_O_4_/activated carbon)	VSM M_Max_ (emu/g)	Kinetic	Isotherm	S_BET_ (m^2^/g)	Heavy metals	Ref
Magnetic pinecone (MNP-PCP)	37.50	Pseudo-second-order	Langmuir	–	Cr(VI)	^38^
(Fe3O4/AC) sunflower head waste	52.2 (Am^2^/kg)	Pseudo-second-order	Langmuir	51.1	{Cr(VI) Cu(II) Cd(II)}	^45^
Fe_3_O_4_-BAC	22.00	Pseudo-first order	Freundlich	–	Cr(VI)	^26^
Fe_3_O_4_/AC from termite feces	–	Pseudo-second-order	Sips	699	Cr(VI)	^30^
Fe_3_O_4_/AC from alga	12.04	Pseudo-second-order	Freundlich	–	Cr(VI)	^24^
(Fe-MCNs) magnetic mesoporous carbon nanospheres	–	Pseudo-second-order	Langmuir	619	Cr(VI)	^37^
Fe_3_O_4_/C-EG core–shell	51.1	Pseudo-second-order	Langmuir	69.70	Cd(II)	^47^
Fe_3_O_4_/C	30.34	Pseudo-second-order	Freundlich	759	Cr(VI)	This study

## Conclusion

In this study, with Fe_3_O_4_/C nanocomposite were synthesized by co-precipitation method using activated carbon derived from vine shoots. The composite was used for Cr removal from simulated wastewater. The different parameters were studied and the results revealed that the removal of hexavalent Chromium is more favorable in an acidic environment with optimal pH of 3. The maximum adsorption capacity of 305.3 mg/g was obtained at the optimal pH and by applying an electric field, it was found that the nanoparticles well follow the Freundlich isotherm properly, and also the adsorption process follows the pseudo-second-order model. The temperature dependent adsorption process showed that, with the increase of bath temperature, the adsorption efficiency increases. The obtained thermodynamic parameters indicate the spontaneity of the adsorption. The high adsorption efficiency and capacity beside ease of separating the nanocomposite from the liquid phase are advantages of the prepared nanocomposite. With major focus on eco-friendly remediation, viable, and cost-efficient methods for THMs (toxic heavy metals), research is still largely in its developmental stage and continuous efforts are in progress worldwide for collecting sufficient information to enable consideration and overcome inherent shortcomings. However, no individual remediation method has been identified that is universally effective and applicable for complete detoxification of the contamination of heavy metals. It can be used as a suitable absorbent for water and wastewater. Among the advantages of this adsorbent, we can mention its catalytic properties, quick and easy separation, as well as not using expensive and long-term filtration and centrifugation methods to separate it from aqueous.

## Materials and methods

### Purchased materials

All chemicals and reagents were of analytical grade; potassium dichromate salt (K_2_Cr_2_O_7_, 99%), (NaOH, 99%), (HCl, 37%), Ferric chloride (FeCl_3_⋅6H_2_O, 99%) and Ferrous chloride (FeCl_2_ 0.4H_2_O, 99%), Zinc chloride (ZnCl_2_, 99%) were purchased from Merck company.

### Experimental procedure of activated carbon

**Figure 15:**
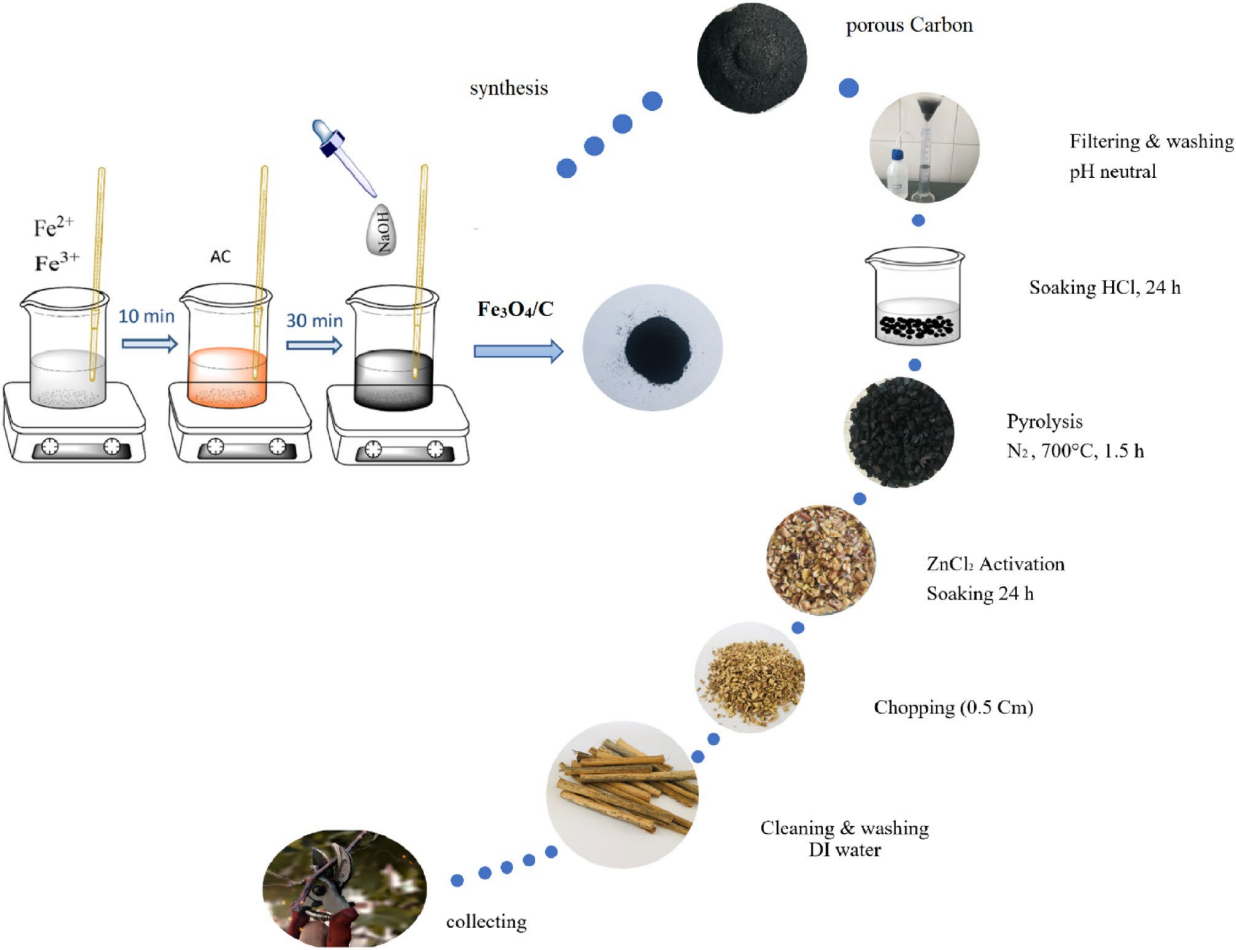
Schematic illustration of the fabrication of vine shoots porous carbon and synthesis of Fe_3_O_4_/C nanocomposite.

Figure 15 is a schematic trend of composite preparation. In the preparation of activated carbon, vine shoots were applied as biomass. The vine shoots were collected after the grape harvest from the vineyard in Urmia city. We confirm that the use of vine shoots in the present study complies with international and national guidelines.

To produce activated carbon from the vine shoots, first they were cleaned manually, followed by washing with deionized water (DI) to remove the dust. After washing, the vine shoots were dried for 3 days, and then the dried biomass was chopped into small pieces (0.5 cm). After that, vine shoots were soaked in ZnCl_2_ solution for 24 h. The saturated samples were dried in an oven at 110 °C for 12 h. The samples were carbonized at 700 °C under nitrogen atmosphere. After 1.5 h of heating, the obtained carbonized material was allowed to cool down gradually for 24 h. The sample was soaked in HCl; the mixtures were left overnight at room temperature and then filtered and washed with deionized water until the pH of filtrate reached 7. As-prepared samples were washed with deionized water twice and then dried at 110 °C for 5 h to become black products. Finally, the activated product was ground by using a high-speed grinder. Then the powders were passed through a 125 μm mesh. Finally, the obtained uniform powders were washed with water and dried at 120 °C to get the activated carbon.

### Synthesis of Fe_3_O_4_/C nanocomposite

We used the co-precipitation method to synthesize Fe_3_O_4_ and Fe_3_O_4_/C nanocomposite. For Fe_3_O_4_/C nanocomposite, first, we prepared an aqueous mixture of 200 ml containing 0.2 mol/L, FeCl_3_⋅6H_2_O and 0.1 mol/L, FeCl_2_ 0.4H_2_O with a molar ratio of Fe^3+^:Fe^2+^ = 2:1. The solution was stirred for 10 min at 80 °C. Subsequently, required molar proportion (1:1) of activated carbon was added to the above solution under constant stirring for 30 min at 80 °C. After that, 50 mL of sodium hydroxide (NaOH) solution with a concentration of 3.4 M was added drop-wise to the mixture and stirred for 30 min and then were carefully added to the above solution (Fig. 15). Afterward, the black powder was filtered and washed with deionized water. Finally, the final product was dried at room temperature for 24 h.

### Characterization methods

Crystal structures and phases in each sample were corroborated by X-ray diffraction (XRD-China; Asenware with AW-XDM300). The morphology of the nanoparticles was studied by scanning electron microscopy FESEM model TESCAN–MIRA 3 equipped with an energy-dispersive X-ray spectroscopy (EDX). The Fourier transform infrared spectra were performed using a FTIR-Jasco, model 680 Plus, at ambient temperature and in the range of 400–4000 cm^−1^. The magnetization hysteresis loops were analyzed by a vibration sample magnetometer (VSM- Meghnatis daghigh kavir Co. Iran) at 300 K. Thermogravimetric analysis (TGA) is the measuring the mass variation of a sample as a function of temperature. The changes in the mass of activated carbon and Fe_3_O_4_/C nanocomposite as a function of temperature in a defined and controlled environment from 25 to 1000 °C were measured by TGA/DTG curves in N_2_ atmosphere at a heating rate of 10 °C min^−1^. The measurements were carried out using a NETZSCH STA 409 PC/PG, Germany. The pore characteristic of the samples was studied by Brunauer–Emmett–Teller (BET) method via nitrogen adsorption–desorption measurements. An atomic absorption spectrophotometer (AAS- Analytik Jena factory, model novaAA 400) was used to determine the concentration of chromium in the solution.

## Data Availability

The datasets used and/or analysed during the current study available from the corresponding author on reasonable request.
